# A Population-Based Multigenerational Family Coaggregation Study of Severe Infections and Obsessive-Compulsive Disorder

**DOI:** 10.1016/j.biopsych.2024.09.004

**Published:** 2024-09-14

**Authors:** Josep Pol-Fuster, Lorena Fernández de la Cruz, Jan C. Beucke, Eva Hesselmark, James J. Crowley, Elles de Schipper, Brikell Isabell, Zheng Chang, Brian M. D’Onofrio, Henrik Larsson, Paul Lichtenstein, Ralf Kuja-Halkola, David Mataix-Cols

**Affiliations:** Centre for Psychiatry Research, Department of Clinical Neuroscience, Karolinska Institutet, Stockholm, Sweden; Stockholm Health Care Services, Region Stockholm, Stockholm, Sweden (JP-F, LFdlC, JCB, EH, JJC, EdS, DM-C); Centre for Psychiatry Research, Department of Clinical Neuroscience, Karolinska Institutet, Stockholm, Sweden; Stockholm Health Care Services, Region Stockholm, Stockholm, Sweden; Centre for Psychiatry Research, Department of Clinical Neuroscience, Karolinska Institutet, Stockholm, Sweden; Stockholm Health Care Services, Region Stockholm, Stockholm, Sweden; Institute for Systems Medicine, Department of Human Medicine, MSH Medical School Hamburg, Hamburg, Germany; Centre for Psychiatry Research, Department of Clinical Neuroscience, Karolinska Institutet, Stockholm, Sweden; Stockholm Health Care Services, Region Stockholm, Stockholm, Sweden; Centre for Psychiatry Research, Department of Clinical Neuroscience, Karolinska Institutet, Stockholm, Sweden; Stockholm Health Care Services, Region Stockholm, Stockholm, Sweden; Department of Genetics, University of North Carolina at Chapel Hill, Chapel Hill, North Carolina; Centre for Psychiatry Research, Department of Clinical Neuroscience, Karolinska Institutet, Stockholm, Sweden; Stockholm Health Care Services, Region Stockholm, Stockholm, Sweden; Department of Medical Epidemiology and Biostatistics, Karolinska Institutet, Stockholm, Sweden; Department of Global Public Health and Primary Care, University of Bergen, Bergen, Norway; Department of Biomedicine, Aarhus University, Aarhus, Denmark; Department of Medical Epidemiology and Biostatistics, Karolinska Institutet, Stockholm, Sweden; Department of Medical Epidemiology and Biostatistics, Karolinska Institutet, Stockholm, Sweden; Department of Psychological and Brain Sciences, Indiana University, Bloomington, Indiana; Department of Medical Epidemiology and Biostatistics, Karolinska Institutet, Stockholm, Sweden; School of Medical Sciences, Örebro Universitet, Örebro, Sweden; Department of Medical Epidemiology and Biostatistics, Karolinska Institutet, Stockholm, Sweden; Department of Medical Epidemiology and Biostatistics, Karolinska Institutet, Stockholm, Sweden; Centre for Psychiatry Research, Department of Clinical Neuroscience, Karolinska Institutet, Stockholm, Sweden; Stockholm Health Care Services, Region Stockholm, Stockholm, Sweden; Department of Clinical Sciences, Lunds Universitet, Lund, Sweden

## Abstract

**BACKGROUND:**

Postinfectious autoimmune processes have been proposed as potential causal risk factors for obsessive-compulsive disorder (OCD). In this large population-based study, we aimed to clarify the familial coaggregation pattern between severe infections and OCD across clusters of relatives with varying degrees of relatedness.

**METHODS:**

We identified 4,916,898 individuals born in Sweden between 1960 and 2008 and followed them until the end of 2020. Each individual was linked to their first-, second-, and third-degree relatives, including monozygotic and dizygotic twins, mothers, fathers, full siblings, maternal and paternal half siblings, aunts, uncles, and cousins. OCD and infection diagnoses from inpatient and specialized outpatient settings were retrieved from the Swedish National Patient Register. We compared the risk of OCD in relatives of probands with severe infections to those of probands without severe infections. Cox proportional hazard regression models, incorporating time-varying exposures, were used to estimate hazard ratios. Dose-response associations were examined using logistic regression models.

**RESULTS:**

Relatives of probands with severe infections had a higher risk of OCD, which increased with genetic relatedness, with hazard ratios (95% CI) ranging from 1.46 (1.07–1.98) in monozygotic twins to 1.10 (1.09–1.11) in cousins. The results remained robust after adjusting for severe infections among relatives, OCD in probands, and comorbid autoimmune disorders in both probands and relatives. A dose-response association was observed between the number of infections in the probands and their odds of OCD, as well as in their relatives.

**CONCLUSIONS:**

The results strongly suggest that the association between severe infections and OCD may be largely driven by shared genetic factors.

Obsessive-compulsive disorder (OCD) is a familial disorder (1,2), with twin-based heritability estimates around 50% (3,4). In recent years, there has been rapid progress in the discovery of common and rare genetic variants that confer risk for the disorder (5,6), but much less is known about potential environmental risk factors (7,8).

Infections have long been hypothesized to be causally associated with OCD through postinfectious autoimmune processes (9–11). While multiple clinical and population-based studies have reported significant associations between childhood infections and OCD (12,13), the idea that infections play a causal role has been questioned. For example, in a Swedish population-based sibling-controlled study (*N* = 2,949,080), severe infections requiring hospitalization before age 3 years were significantly associated with increased risk of developing OCD (hazard ratio [HR], 1.19; 95% CI, 1.14–1.25), but these associations were no longer statistically significant when individuals affected with OCD were compared with their unaffected full siblings (HR, 0.93; 95% CI, 0.85–1.01) (13).

Genetic factors are widely recognized to influence an individual’s susceptibility to infections (14,15). Consequently, it is plausible that genes that contribute to infection susceptibility may also contribute to OCD liability (genetic pleiotropy) (13). This notion is supported by a population-based study that found the association between severe infections and psychiatric disorders to be at least partially genetically driven, although OCD was not specifically examined (15). In a report from the OCD Collaborative Genetics Association Study, first-degree relatives (*N* = 1045) of individuals with OCD had higher rates of self-reported infections than the expected population prevalence in the United States, independent of OCD status (16). However, this study was limited by a small sample, retrospective self-report, incomplete coverage of relatives, and the lack of a control group. Thus, whether severe infections and OCD coaggregate in families remains an open question.

The aim of this study was to estimate the familial coaggregation between severe infections and OCD in a Swedish total population cohort. We hypothesized that 1) there would be a significant association between severe infections and OCD at the population level, 2) the biological relatives of individuals with severe infections would have higher risks of OCD than relatives of individuals without severe infections, and 3) the strength of the associations would increase together with genetic relatedness.

## METHODS AND MATERIALS

### Data Sources

We used the unique Swedish personal identification number (17) to link several health and administrative registers. The Total Population Register (18), which provides information on emigration and immigration from and to Sweden since 1961 and 1969, respectively, was used to identify the study cohort and obtain information on migration. The Cause of Death Register (19), which contains information on dates and causes of all deaths since 1961, was used to obtain information on deaths. The Multi-Generation Register, which contains information about the kinship of each person registered in Sweden from 1960 and those who were born since 1932, was used to identify biological kinships (20). The Swedish Twin Registry (21), which contains data on all twins born in Sweden since 1886, was used to retrieve zygosity information. The National Patient Register (NPR) (22), which covers all inpatient hospital admissions since 1969 and outpatient specialist care since 2001, was used to obtain data on clinical diagnoses.

### Study Population

The study population consisted of all individuals born in Sweden between January 1, 1960, and December 31, 2008, with available information on both biological parents. Individuals were excluded if they emigrated or died before 1987 or before age 6 years, or if they were diagnosed with OCD before 1987 (when the coverage of inpatient care is complete in the NPR).

We used individuals in this cohort (probands) to identify proband-relative pairs. We linked each proband with their biological relatives and constructed 10 subcohorts of relatives: monozygotic (MZ) twins, dizygotic (DZ) twins, mothers, fathers, full siblings, maternal half siblings, paternal half siblings, aunts, uncles, and cousins.

The subcohorts of MZ and DZ twins, full siblings, maternal and paternal half siblings, and cousins were entirely composed of proband-relative pairs with relatives included in the study cohort. However, the subcohorts of mothers, fathers, aunts, and uncles could include proband-relative pairs with relatives from outside the study cohort. To ensure a similar birth year range and mitigate significant generational differences in these subcohorts, we excluded any proband-relative pairs from the analysis where the relatives were born before 1940 or after 1990. We also excluded proband-relative pairs from these subcohorts that contained relatives who died, emigrated, or were diagnosed with OCD before 1987.

Probands in the cohort and relatives in the subcohorts were followed from birth or from January 1, 1987, whichever came last, until the date of OCD diagnosis, emigration, death, or the end of the follow-up (December 31, 2020), whichever came first.

### Exposure: Severe Infections

We defined severe infections (viral, bacterial, or unknown) as the first recorded infection in the NPR, including both inpatient (from 1987) and specialized outpatient settings (from 2001) (see ICD codes in Table S1). In the within-individual analysis, probands were considered unexposed before their first diagnosis of severe infections and exposed thereafter. Relatives were considered unexposed prior to the proband’s date of first diagnosis of severe infections and exposed thereafter.

### Outcome: OCD Diagnosis

We retrieved the first instance of a recorded OCD diagnosis (ICD-8 code: 300.3; ICD-9 code: 300D; ICD-10 code: F42) after the age of 6 from the NPR to limit the risk of diagnostic misclassification (23,24). The ICD codes for OCD in the NPR have shown excellent interrater reliability (kappa = 0.98) and moderate to excellent validity (positive predictive value in ICD-8 = 0.55, ICD-9 = 0.64, and ICD-10 = 0.91–0.96) (25).

### Statistical Analysis

First, we analyzed the within-individual association between severe infections and OCD in the whole cohort. To that end, we fitted a Cox regression model with attained age as the underlying time scale and severe infections as a time-varying exposure to calculate HRs and 95% CIs. These analyses were adjusted for sex and birth year (coded categorically).

To assess the familial coaggregation of severe infections and OCD, i.e., to examine the risk of OCD in relatives of probands exposed to severe infections compared with relatives of unexposed probands, we performed a series of Cox regression models with attained age as the underlying time scale and with time-varying exposures in each cohort of relatives. These models were adjusted for the sex and birth year of both the proband and the relative in all cohorts, except for MZ twins, DZ twins, mothers, fathers, aunts, and uncles (model 1). In the MZ twin cohort, only the relative’s sex and birth year were adjusted. In the DZ twin cohort, adjustments included the relative’s birth year and sex as well as the proband’s sex. Analyses of mothers, fathers, aunts, and uncles were not adjusted for the relative’s sex. All subsequent models were based on model 1.

Next, we explored whether familial coaggregation was better explained by the direct effect of severe infections by also adjusting for severe infections in the relatives in each relative cluster (model 2). In a third model, we examined whether the results in model 1 were better explained by the co-occurrence of OCD in the proband and the relative by also adjusting for OCD in the proband (model 3). Furthermore, because autoimmune disorders are associated with both proneness to infections (26) and OCD (27), we adjusted for autoimmune disorders in the relatives (model 4) and for autoimmune disorders in the probands (model 5). Autoimmune disorders were defined as the first diagnosis of any autoimmune disorder from the NPR (see ICD codes in Table S1).

Finally, to further confirm the robustness of our findings across different methods, the within-individual analysis and the analyses in models 1, 2, and 3 were repeated using logistic regression models.

A post hoc logistic regression analysis was conducted to examine the association between the number of severe infections in the probands during the study period and their odds of OCD, as well as the odds in their relatives. We categorized the number of infections into 1 infection, 2 infections, and 3 or more infections. To ensure consistency and avoid counting multiple hospital contacts for the same infection (e.g., relapses, multiple visits due to nonremission of symptoms, changes in diagnosis), a new infection was defined as an inpatient or outpatient contact that occurred at least 2 months after the previous infection. These models were adjusted for sex and birth year.

Robust standard errors were applied in all analyses to address familial clustering. Data were analyzed from October 1, 2023, to June 1, 2024. Statistical analyses were conducted using SAS software (version 9.4; SAS Institute) and in R using the Survival (28) and Drgee packages (29).

## RESULTS

### Cohort Characteristics

Descriptive characteristics of the cohort are presented in Table 1. A total of 4,916,898 individuals were included in the cohort. Among them, 1,811,364 (36.8%) were exposed to at least 1 severe infection during the study period. At the end of the follow-up period, 46,018 (0.9%) individuals were diagnosed with OCD. The median age at first OCD diagnosis was 23.8 years (IQR = 17.4–32.1 years), and the median time from exposure to infection and OCD diagnosis was 10.3 years (IQR = 5.2–15.1 years).

### Familial Coaggregation

**Figure 1.**
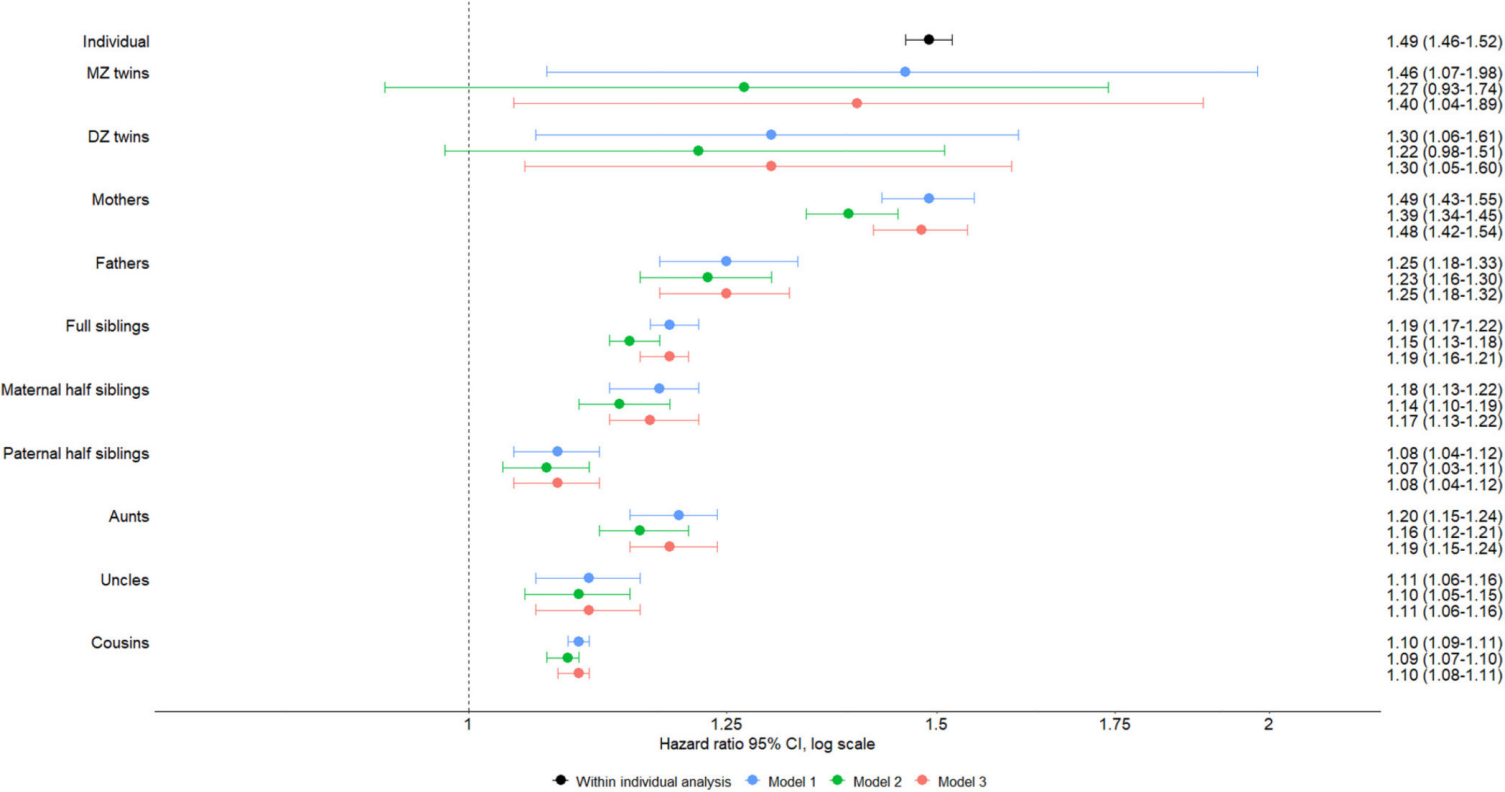
Hazard ratios for obsessive-compulsive disorder in relatives of probands diagnosed with severe infections. The within-individual analysis (black) was adjusted for sex and birth year (categorical). Model 1 (blue) examined the risk of obsessive-compulsive disorder for relatives of probands with severe infections, adjusted for the proband’s and relative’s sex and birth year (categorical). Model 2 (green) was based on model 1, also adjusting for severe infections in the relatives. Model 3 (red) was based on model 1, also adjusting for obsessive-compulsive disorder in the probands. DZ, dizygotic; MZ, monozygotic.

Table 2 shows the total number of individuals included in the analyses for each cluster of relatives. Figure 1 depicts within-individual associations and familial coaggregation patterns between severe infections and OCD. Individuals diagnosed with severe infections had a significantly increased risk of OCD (HR, 1.49; 95% CI, 1.46–1.52) (Figure 1).

Biological relatives of individuals with infections had increased risk of OCD, and the risk increased together with the degree of genetic relatedness (model 1 in Figure 1). First-degree relatives had the highest estimates: MZ twins (HR, 1.46; 95% CI, 1.07–1.98), DZ twins (HR, 1.30; 95% CI, 1.06–1.61), mothers (HR, 1.49; 95% CI, 1.43–1.55), fathers (HR, 1.25; 95% CI, 1.18–1.33), and full siblings (HR, 1.19; 95% CI, 1.17–1.22). Second-degree relatives tended to have higher estimates than third-degree relatives: maternal half siblings (HR, 1.18; 95% CI, 1.13–1.22), paternal half siblings (HR, 1.08; 95% CI, 1.04–1.12), aunts (HR, 1.20; 95% CI, 1.15–1.24), uncles (HR, 1.11; 95% CI, 1.06–1.16), and cousins (HR, 1.10; 95% CI, 1.09–1.11).

The significantly larger estimates observed in mothers than in fathers and in maternal half siblings than in paternal half siblings, as indicated by the non-overlapping CIs, suggested potential maternal effects. Therefore, we further investigated the risk among aunts and uncles, comparing those from the maternal and paternal sides. No statistically significant differences were found between maternal (HR, 1.17; 95% CI, 1.13–1.21) and paternal (HR, 1.14; 95% CI, 1.10–1.19) sides, as indicated by the overlapping CIs.

The familial clustering patterns persisted after adjusting for severe infections in the proband’s relatives, with a slight attenuation of the estimates (model 2 in Figure 1). Similarly, no major changes in the results were observed after adjusting for OCD in the probands (model 3 in Figure 1) and accounting for comorbid autoimmune disorders in both the proband and their relatives (models 4 and 5 in Figure S1). The complementary logistic regression analyses consistently showed a familial coaggregation pattern (models 1, 2, and 3 in Table S2).

### Post Hoc Analyses

**Figure 2.**
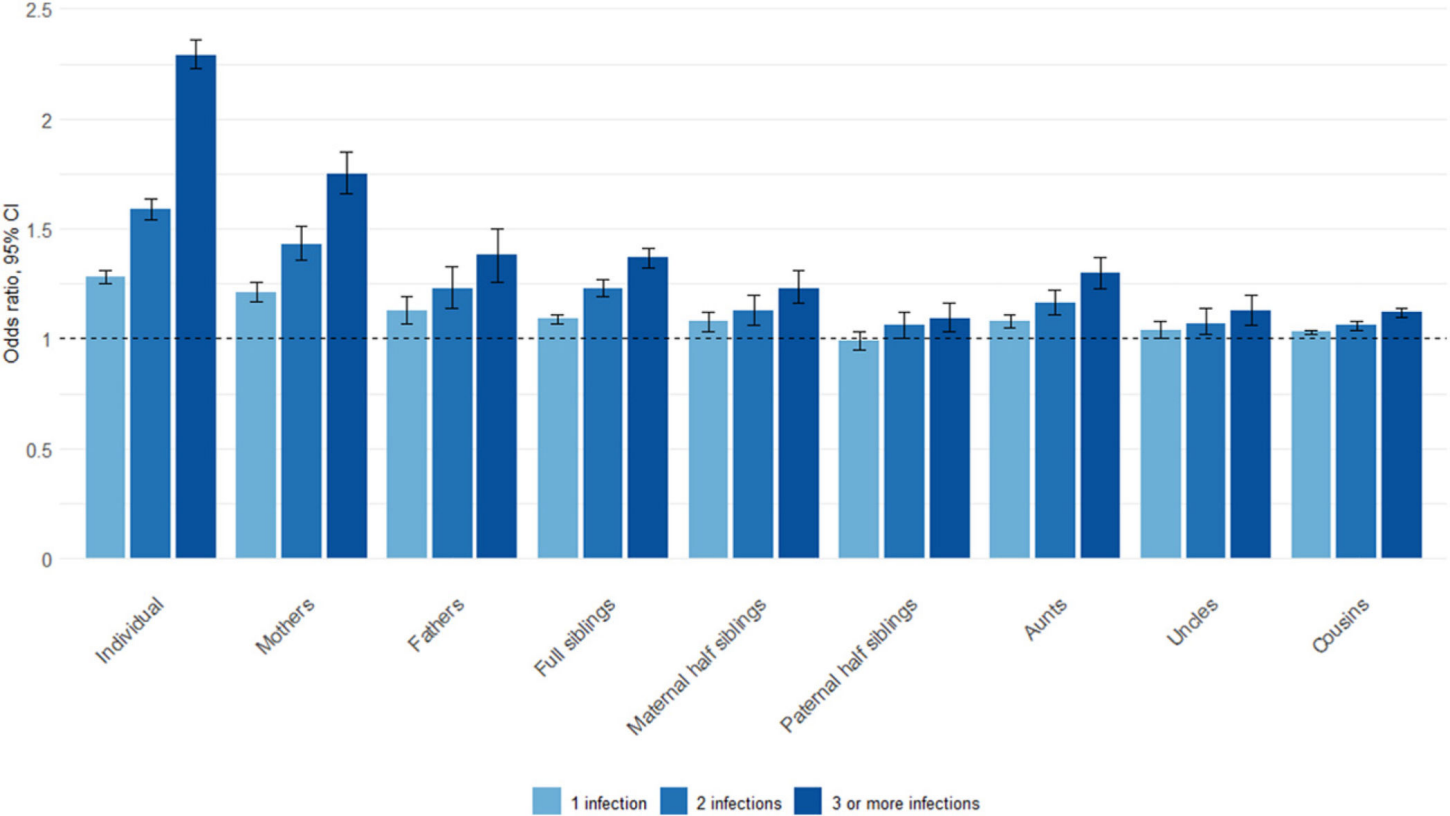
Odds ratios for obsessive-compulsive disorder in relatives of probands diagnosed with 1, 2, and 3 or more severe infections. The within-individual analysis was adjusted for sex and birth year (categorical). The analyses in the relatives were adjusted for the proband’s and relative’s sex and birth year (categorical).

A dose-response association was observed between the number of infections in the proband and the odds of an OCD diagnosis in the same individuals and in their relatives (Figure 2 and Table S3). Compared to individuals without any record of infection, the odds of OCD increased by 28% (odds ratio, 1.28; 95% CI, 1.25–1.31) for those diagnosed with 1 infection, by 59% (odds ratio, 1.59; 95% CI, 1.54–1.64) for those diagnosed with 2 infections, and by 129% (odds ratio, 2.29; 95% CI, 2.23–2.36) for those diagnosed with 3 or more infections. A similar gradient was observed in the various groups of relatives wherein the relatives of probands diagnosed with 3 or more infections had the highest odds of OCD (Figure 2 and Table S3).

## DISCUSSION

In this population-based, multigenerational family coaggregation study, we found that severe infections were associated with OCD, both within individuals and across biological relatives, suggesting an important contribution of familial factors to the observed association. The observed coaggregation remained robust even after adjusting for severe infections among relatives, OCD in the probands, and comorbid autoimmune disorders in both probands and relatives. Our results were also robust to various analytical approaches. To the best of our knowledge, this study is the first to show that severe infections and OCD coaggregate within the same families.

The observed coaggregation pattern, with a clear risk gradient associated with closer genetic relatedness, strongly suggests a significant role of genetic factors. This genetic influence is also evidenced by the similar risks observed in fathers and in full siblings (both sharing 50% of their genes with the probands but the latter sharing a larger proportion of their environment). Likewise, we observed similar risks in aunts and in maternal half siblings (both sharing 25% of their genes but the latter sharing a larger proportion of their environment). The increased risks observed in aunts and uncles (sharing 25% of their genes with the probands and 0% of their environment) and cousins (sharing 12.5% of their genes and 0% of their environment) further support a genetic component. Additionally, a post hoc analysis showed a clear dose-response association, which reinforces the idea that proneness to severe infections and OCD share genetic risk factors.

The higher risk in mothers than in fathers and in maternal half siblings than in paternal half siblings may suggest maternal genetic effects, which have previously been proposed for OCD (30); however, this explanation alone does not account for the higher risk observed in aunts than in uncles. Additionally, in our analyses, there were no significant differences between maternal and paternal aunts and uncles. Therefore, the most likely explanation for these results is unknown biases and/or sex effects. Further research is needed to better understand this phenomenon.

The findings of this study are consistent with previous research indicating a genetic correlation between severe infections and psychiatric disorders in general (15). A small exon-focused OCD genome-wide association study showed significant associations in 2 different regions of the major histocompatibility complex locus on chromosome 6 (31), which has a major role in the adaptive immune system and is known to be associated with psychiatric disorders (32). More recently, the largest OCD genome-wide association study reported to date found a genome-wide significant locus in the major histocompatibility complex, and gene-based tests further revealed 4 causal genes in this locus (5). Thus, it is plausible that individuals with variants in these genes may carry a propensity to infections, autoimmune disorders, and OCD, potentially via genetic pleiotropy (13).

Overall, the results of the current study expand our understanding of the complex relationship between infections and OCD. Various sources of evidence converge to suggest that there may be a small group of individuals with autoimmune-related OCD (11). Our results suggest that, rather than playing a direct causal role, infections may only be one part of the complex clinical picture. Clinically, these complex cases are best managed by multidisciplinary teams that have the capacity to focus on the somatic aspects of the condition while simultaneously prioritizing evidence-based treatments for OCD (11).

### Strengths and Limitations

To our knowledge, this was the first study to analyze the familial coaggregation of severe infections and OCD in a large population-based cohort with longitudinally collected data, which minimizes the risk of selection and recall bias. The Multi-Generation Register made it possible to identify different clusters of relatives with different degrees of relatedness (20). Moreover, the diagnostic codes for OCD have been validated previously (25).

Some limitations of this study should be considered. First, power was limited when using twins in our analyses. Nonetheless, the overall results and conclusions were well supported by the inclusion of other relatives. Second, our results are based on patients diagnosed in specialist services who actively sought medical help. This means that individuals with milder infections or individuals with OCD diagnosed by general practitioners may be less well covered by our registers. Additionally, outpatient OCD diagnoses have only been registered since 2001, leading to incomplete coverage before that period and potentially including more severe cases prior to 2001.

## Conclusions

This study showed a clear familial coaggregation between severe infections and OCD across clusters of relatives, with higher degrees of genetic relatedness linked to stronger coaggregation. The results are strongly suggestive of a shared genetic liability to both proneness to severe infections and OCD.

## Supplementary Material

Supplementary Information

## Figures and Tables

**Table 1.** Distribution of Study Cohort Characteristics

	Severe Infections, *n* = 1,811,364	No Infection, *n* = 3,105,534
OCD		
No record of OCD	1,788,756 (98.8%)	3,082,124 (99.2%)
OCD	22,608 (1.2%)	23,410 (0.8%)
Sex		
Female	1,021,329 (56.4%)	1,369,817 (44.1%)
Male	790,035 (43.6%)	1,735,717 (55.9%)
Birth Year		
1960−1969	316,270 (17.5%)	740,138 (23.8%)
1970−1979	323,840 (17.9%)	663,279 (21.4%)
1980−1989	377,873 (20.9%)	591,940 (19.1%)
1990−1999	407,440 (22.5%)	620,605 (20%)
2000−2008	385,941 (21.3%)	489,572 (15.8%)

Values are presented as *n* (%).

OCD, obsessive-compulsive disorder.

**Table 2.** Number of Individuals in the Study Cohort and Each Cluster of Relatives

Total Cohort and Family Clusters	Unique Individuals	Unique Pairs^a^	Observations^b^	Excluded
Total Cohort	4,916,898	NA	4,916,898	NA
MZ Twins	20,652	10,326	20,604	4,896,246^c^
DZ Twins	46,136	23,068	46,081	4,870,762^c^
Mothers	4,174,648	4,174,648	4,174,648	742,250^d^
Fathers	3,867,964	3,867,964	3,867,964	1,048,934^d^
Full Siblings	3,771,371	2,909,905	5,819,810	1,145,527^e^
Maternal Half Siblings	684,297	530,848	1,061,696	4,232,601^e^
Paternal Half Siblings	756,466	644,063	1,288,126	4,160,432^e^
Aunts	3,000,458	5,599,160	5,599,160	1,916,440^f^
Uncles	3,086,815	5,912,738	5,912,738	1,830,083^f^
Cousins	3,715,924	11,591,438	23,182,876	1,200,974^g^

In all pairs of twins, siblings, and cousins, each individual contributed to the analysis at least once, with information on exposure and on outcome.

DZ, dizygotic; MZ, monozygotic; NA, not applicable.

a Number of the unique pairs identified (e.g., mother-offspring, sibling 1−sibling 2).

b Number of observations included in the analysis (i.e., all possible combinations of pairs in which members contributed to the analysis with information on exposure and outcome).

c Probands with no MZ or DZ twins identified from the study cohort.

d Probands whose parents were born before 1940 or after 1990 or died, emigrated, or were diagnosed with obsessive-compulsive disorder before 1987.

e Probands with no siblings of a certain degree of relatedness identified from the study cohort.

f Probands whose uncles/aunts were born before 1940 or after 1990, whose uncles/aunts were twins of their parents, or died, emigrated, or were diagnosed with obsessive-compulsive disorder before 1987.

g Probands with no cousins identified from the study cohort or if parents of cousins are twins.
